# Editorial: Appropriateness and safety of using cannabinoid and psychedelic medicines as treatments for psychiatric disorders

**DOI:** 10.3389/fpsyt.2023.1191970

**Published:** 2023-05-12

**Authors:** Grace Blest-Hopley, Ben H. Amit, Aisling O'Neill, Simon G. D. Ruffell

**Affiliations:** ^1^King's College London, London, United Kingdom; ^2^Tel Aviv Medical Center, Tel Aviv, Israel; ^3^Department of Psychology, University College Dublin, Dublin, Ireland; ^4^Department of Psychology, St Patrick's Mental Health Services, Dublin, Ireland; ^5^Onaya Science, Iquitos, Peru; ^6^Psychae Institute, Melbourne, VIC, Australia; ^7^School of Population and Global Health, University of Melbourne, Melbourne, VIC, Australia

**Keywords:** psychedelics, cannabis, cannabinoids, hallucinogenic, psilocybin

Medical cannabis and psychedelics have shown promising results in treating various psychiatric disorders (1), but the lack of robust evidence remains a significant challenge. In this edition, we share intriguing insights into the specific applications of certain substances. However, it's important to note the current dearth of evidence and insufficient means of data collection. This lack of information can hinder the progress of developing and utilizing these substances as potential therapeutics, underscoring the urgent need for further research.

In summary of the papers reported in the edition; Colizzi et al. systematic review explored the potential of palmitoylethanolamide (PEA) in disrupting mechanisms underlying neurodegeneration, and the authors concluded that PEA may act as a modifying agent in the early stages of neurocognitive disorder, preventing neurodegeneration via endogenous repair processes (Colizzi et al.).

Nacasch et al. reported that medical cannabis improved symptoms, especially sleep, in treatment-resistant combat PTSD (Nacasch et al.). Schlag et al. commented on the need for real-world evidence when assessing the effectiveness and safety of medical cannabis. They argued that using real-world evidence is essential when considering the complex, multi-molecule compounds that are often used in a population with complex and co-morbid conditions, excluding them often from RCTs. This paper provides a framework for ensuring consistent and quality analysis of such real-world evidence, which can highlight therapeutic potential for further investigation in more rigorous RCTs and inform the clinical use of these substances in the real world (Schlag et al.).

Van der Meer et al. showed the potential of psilocybin in treating substance use in combination with psychotherapy. While all the studies they reviewed showed psilocybin had a positive effect on substance abuse, they also highlighted the need for more studies with larger participant numbers to find the “best practices” for treating substance abuse (Van der Meer et al.). A naturalistic of psychedelics and substance use found that psychedelics that are part of more ceremonial practices were associated with lower levels of substance use, but not psychedelics that are more commonly used recreationally (Rabinowitz et al.). MacCallum et al. report on psilocybin outlined mechanisms and use cases for the substance and emphasized the importance of caution and risk mitigation in future studies and real-world use (MacCallum et al.).

The common themes in these papers highlight the need for more research to make robust claims on the efficacy of cannabinoid and psychedelic substances for psychiatric disorders. One of the key themes is the utilization of real-world evidence to identify therapeutic areas that can be explored in rigorously designed trials, but also practical considerations for appropriateness, efficacy, and safety in a population.

Moving forward, with the ever-growing body of work on cannabinoids and psychedelics, there is a need to shift focus toward specific groups, such as women and the elderly suffering from neurodegenerative disorders. Group-specific studies that investigate the unique biology of these groups have traditionally been under-studied, leading to a lack of understanding of how these substances might work for different populations. Therefore, it is essential to better understand the safety and efficacy of these substances in different groups to define who these treatments could help, and when they may not be appropriate and could even cause harm.

This is particularly relevant when considering adolescent populations. These forms of treatment are often over-inflated in their potential and under-studied in practice, sending potentially harmful messages about their safety to the wider society. As many of these substances have been used recreationally, the decriminalization and legalization of these substances have led to an increasing interest in their therapeutic potential. Therefore, it is crucial to emphasize caution and risk mitigation in future studies and real-world use of such substances.

In addition, research should explore how these substances affect regions other than the central nervous system (CNS) and whether these effects are always helpful. As the psychedelic renaissance coincides with a boom in cannabis and cannabinoid research, traditional randomized controlled trials (RCTs) are not always appropriate due to biasing around blinding and inter-study participant influences, making it difficult for the field to ignore. Rigorous scientific exploration of the true mechanisms and safety around these substances is still in its infancy. The field would benefit from more complex scientific modeling in light of the many cofounders to this traditional research approach. Therefore, researchers, regulators, and the interested wider population should be cognizant of this need.

Lastly, there is a clear need for much more research in these areas. Although there is ongoing research, it is often carried out by commercial companies, making the data unavailable. Ideally, more large-scale RCTs using a variety of substances and techniques will allow for comparative analysis and identifying the right treatment for the right person. This will help to establish a robust evidence base for the use of these substances in the treatment of psychiatric disorders and neurodegenerative disorders, allowing for informed clinical decision-making and better patient outcomes.

## Author contributions

The editorial was contributed to by all editors and finalized by GB-H. All editors reviewed and approved the final manuscript.
